# Common features of environmentally and socially engaged community programs addressing the intersecting challenges of planetary and human health: mixed methods analysis of survey and interview evidence from creative health practitioners

**DOI:** 10.3389/fpubh.2025.1449317

**Published:** 2025-01-27

**Authors:** Linda J. M. Thomson, Ailsa Critten, Victoria Hume, Helen J. Chatterjee

**Affiliations:** ^1^Department of Biosciences, University College London, London, United Kingdom; ^2^Department of Arts and Sciences, University College London, London, United Kingdom; ^3^The Culture, Health and Wellbeing Alliance CIC, Barnsley, United Kingdom

**Keywords:** community programs, creative health, human health, mental health, planetary health, social determinants of health, sustainability, wellbeing

## Abstract

Depending on environmental and social determinants, planetary health impacts unequally on human health. As it is likely that creativity and culture are under-tapped resources, the potential to address community and environmental issues to tackle health inequalities, especially those resulting from climate injustice, has not yet been fully realized. The study aimed to identify common features of environmentally and socially engaged UK community programs addressing the intersecting challenges of planetary and human health. A short survey was used to screen participants for in-depth semi-structured interviews. Inclusion criteria comprised adult practitioners offering environmentally and socially engaged community programs of creative and cultural activities leading to health and environmental outcomes. Thematic analysis of 19 surveys and eight interviews identified 146 responses, from which 12 themes with 98 subthemes were derived. Seventy per cent of responses were distributed across five major themes: ‘Collaboration and partnerships’, ‘Community health and wellbeing’, ‘Connection to nature’, ‘Funding’ and ‘Mental health’. Within these five themes, 10 subthemes which resulted from three or more similar responses by different participants were deemed common features of community programs. Two of the 10 subthemes: ‘Connection to nature in children’ and ‘Relationship with natural world’ within the major theme: ‘Connection to nature’ addressed planetary and human health directly through practices recognizing environmental and human interdependency. Four of the 10 subthemes: ‘Influencing wider systems’ within the major theme ‘Collaboration and partnerships’; and ‘Looking after our staff’, ‘Preventative measures’ and ‘Research evidence’ within the major theme ‘Mental health’; addressed planetary and human health indirectly through practitioner partnership influence over policies relating to climate change and by addressing concern for the environment manifesting in eco-anxiety. The study indicates the need for inclusive practice, partnership work, and sustainable funding that can support practitioner wellbeing and the process, outputs and impacts of natural and sustainable environment-based health interventions and other resources instrumental in preventative healthcare.

## 1 Introduction

Human and planetary health are “inextricably linked” [(1), p. 1] and “mutually reliant” [(2), p. 1], consequently, the “need to focus on creating healthy societies on a healthy planet” is becoming increasingly apparent [(1), p. 1]. The United Kingdom Research and Innovation (UKRI) Planetary Health Network has agreed a working definition of ‘planetary health’ based on that of the Rockefeller Foundation–Lancet Commission on Planetary Health (3), which “focuses on the health impacts (in humans, animals, and plants) of human-caused disruptions of Earth’s natural systems and the development and evaluation of potential actions to create positive feedback loops between a healthy environment and a healthy society” [(4), p. 2]. Ill health and premature death, for example, have been associated with pollution (5). Planetary health has been described as a “solutions-oriented, transdisciplinary field and social movement” that seeks to address such disruptions to Earth’s natural systems [(6), p. 1]. Planetary health is therefore premised on the idea that the wellbeing of humanity and the earth are interdependent, and a sustainable world is one in which “the earth thrives and people can pursue flourishing lives” [(7), p. 3].

The notion of planetary health arose from environmental movements of the 1970s and 1980s (8). In 1980, for example, Friends of the Earth, extended the World Health Organization (WHO) definition of health– “a state of complete physical, mental and social wellbeing” [(9), p. 1]—“a state of complete physical, mental, social and *ecological* well-being—personal health involves planetary health” [(10), p. 9]. Since the Rockefeller Foundation-Lancet Commission (3), this concept has been increasingly mainstreamed in clinical literature (8). The Commission determined that “human civilization… now risks substantial health effects from the degradation of nature’s life support systems in the future… driven by highly inequitable, inefficient, and unsustainable patterns of resource consumption and technological development, together with population growth” [(3), p. 1973]. A recent review of health inequalities in the UK further states that “economic growth without attending to its environmental impact… is not an option for the country or for the planet” [(11), p. 18]. Sustainability and wellbeing are both central to the ‘planetary boundaries’ framework [(12), p. 1], which describes the environmental limits in which humans can safely operate “without endangering the vitality of the ecosystems” [(13), p. 169].

Allied to planetary health are older concepts of ‘one health’ (14) and ‘holistic health’ (15). The one health approach has been described as a multi-disciplinary effort at local, national, and global levels “to attain optimal health for people, animals, and our environment” [(16), p. 3]. It gained traction in response to serious zoonotic disease outbreaks (17)—and has been endorsed by international agencies including the WHO, World Organization for Animal Health, and United Nations Food and Agriculture Organization (14). The holistic health approach focuses largely on non-medical treatments—often described as complementary or alternative—and, akin to the WHO definition of health, can be expressed as “complete or total patient care that considers the physical, emotional, social, economic, and spiritual needs of the person” [(15), p. 1935]. The environmental component of one health and holistic health may have been comparatively neglected, however (17). Consequently, recent research has aimed to rebalance the emphasis on the health of people, animals, and ecosystems while recognizing their interdependence (18), and suggests that a one health approach could be applied at community, regional, national, and global scales (19).

Depending on the environmental and social determinants of health—i.e., “the conditions in which people are born, grow, live, work and age and inequities in power, money and resources” [(20), p. 5]—planetary health impacts unequally on human health. The concept of ‘climate injustice’, moreover, speaks to the fact that while poorer nations have contributed far less to climate change, they are likely to suffer disproportionately from its effects (21). The Marmot review into health inequalities avers that “tackling social inequalities in health and tackling climate change must go together” [(11), p. 15]; moreover, that environmental stability “should be a more important societal goal than simply more economic growth” [(11), p. 18]. Yet the WHO Commission reports that health is not prioritized in environmental policies (5). Furthermore, adaptation to climate change means “we must do things differently” and “creating a sustainable future is entirely compatible with action to reduce health inequalities” [(11), p. 18]. Others have argued for shifting the center of our definition of socially determined health: to “for too long, humans alone have been the focus… and it is time to clearly acknowledge and define the determinants of planetary health in a more eco-centric way” [(22), p. E111].

Health inequalities are defined by the NHS as “unfair and avoidable differences in health across the population, and between different groups within society” [(23), p. 1]. Their consequences might include differences in length of life, health conditions and access to care (23). In a study aiming to determine priority areas for future research into inequalities in the UK and methods by which they might be addressed, participants identified health, social care, living standards and economic factors as key areas of inequality which were interconnected and needed to be addressed together “through tackling societal and structural inequalities, chiefly environmental conditions and housing, and having an active prevention program” [(24), p. 11]. According to the Institute of Health Equity, in the period prior to COVID-19 (2011–2019), 890,000 people died earlier in the most deprived quintile of England (11), with some areas of inequality widening during COVID-19 (20, 24).

A significant body of evidence, however, highlights the potential of community assets, such as museums, libraries, galleries, parks and natural heritage, to tackle health inequalities (25). For example, a review of the work of charities, freelance creative practitioners, social enterprises and local authority museums offering online workshops, pre-recorded performances, activity packs, and co-produced exhibitions or artwork during COVID-19, found their intended outcomes included improving institutional wellbeing, tackling loneliness and isolation, and supporting social or family connections (26). A further review of the social impact of music-making interventions showed that intended outcomes covered “areas of wellbeing, inclusion, confidence, empowerment, and co-operation” [(27), p. 117]. It is increasingly acknowledged that public health challenges cannot be undertaken entirely by existing healthcare systems, and practices outside of health might make a greater contribution to population health outcomes than the health sector itself (28). One such practice with potential to influence population health is ‘creative health’ which has developed in part from the broader field of ‘participatory art’ (29). At the roots of participatory art lie a challenge to professional and theoretical silos connecting “art, social work, politics, philosophy, environmentalism, therapy, community development, activism, health, esthetics, social justice and many other fields” [(29), p. 26].

The All-party Parliamentary Group on Arts, Health and Wellbeing (APPGAHW) inquiry report was among the first publications to use ‘creative health’ as an umbrella term for a multitude of creative and cultural practices, and to draw attention to the policy implications of the role of creativity in health and wellbeing (30). The report asserted that “the arts can help meet major challenges facing health and social care” and, in referencing more than a thousand creative projects and research studies, illustrated the impact of creativity and culture on health and wellbeing [(30), p. 154]. Creative health activities include “visual and performing arts, crafts, film, literature, cooking and creative activities in nature, such as gardening” [(31), p. 10].

In addition to arts practices, researchers have found a “significant amount of natural environment-based health intervention activity,” which suggest health outcomes could be improved through, for example “good quality urban greenspace interventions” [(32), p. 81]. Briefings focused on the benefits of exposure to natural environments show evidence of “physical, social, cognitive and emotional development” for children and young people [(33), p. 10–11] and a correlation of connection to nature with “pro-environmental and pro-conservation behavior and wellbeing” [(34), p. 9]. Analysis of data from two surveys assessing the impact of culture-, health- and nature-based engagement on mitigating the adverse effects of public health restrictions during COVID-19 showed that over four fifths of adult respondents participated in activities more often than before the pandemic (35). Although the highest frequency of response was for sport and fitness, other activities in the upper quartile included cooking or baking; crafts, textiles, and decorative arts; gardening or looking after plants; and painting, drawing, printmaking, and sculpture. The authors concluded that engagement with culture, health, and nature-based activities “appeared to improve psychological wellbeing, reduce loneliness and engender feelings of social connectedness” [(35), p. 18].

Recently, a new Creative Health Quality Framework, co-produced with practitioners, recognizes the diversity of creative health practice and recommends that good practice should follow eight principles: person-centered, equitable, safe, creative, collaborative, realistic, reflective, and sustainable. ‘Sustainable’ in this context is defined as, working toward “a positive, long-term legacy for people and planet” [(36), p. 7]. Expanding upon the Creative Health Quality Framework recommendations, the current research aimed to identify the common features of environmentally and socially engaged community programs, specifically those addressing the intersecting challenges of planetary and human health, through analysis of surveys and interviews with creative health practitioners.

## 2 Methods

### 2.1 Design

The qualitative study consisted of an online survey and in-depth interview. The survey comprised five open-ended question requiring brief responses (Table 1). Interviews comprised nine open-ended semi-structured questions under six categories (Table 2). Survey and transcribed interview data were analyzed using deductive thematic analysis informed by an interpretivist epistemological perspective using constructivist research methods. Thematic analysis was conducted in NVivo 1.7 and descriptive statistics in IBM SPSS 28.

**Table 1:** Survey questions.

In your own words, please give a brief description of the context of your work. What would a typical session look like for a participant?
How does your work address multiple aspects of health and wellbeing including the wider determinants of health such as poverty, housing, legal issues, or the environment? Please describe practical tasks or features within your work that address these different issues. *(If referring to a specific project or program of work, please provide relevant links.)*
What advice would you give to another practitioner in this field who is aiming to make their program more socially and environmentally engaged?
Are there any organizations/individuals in this field who you recommend we contact for involvement in this study? *(We are looking to involve creative/cultural practitioners working on health/social/environmental issues.)* Please give details below.
Any further comments? If there’s anything else you’d like to add, please do so below.

**Table 2:** Interview questions.

Categories	Questions
Introduction	Could you summarize your practice and the work that you do? *Prompt: What might a typical session look like for a participant?*
	What outcomes are you hoping for and how do you know if you have achieved them? *Prompt: How do you know if the program is working?*
Community assets	What key resources and spaces does your work make use of and why are they important to your work?
	What is your project’s value/impact within the local community context?
Environment	How do you frame your work in relation to climate change/planetary health? *Prompts: What environmental/planetary issues does your work aim to address?*
Health and wellbeing	What health/wellbeing issues does your work aim to address? What does tackling these issues look like in practice for you?
Social and economic	What social and economic issues does your work aim to address and how does your work tackle them?
Conclusions	Considering these different health, social and environmental aspects of your work, do you feel your work prioritizes any outcomes in particular? *If no, ask to expand on how these different areas are prioritized equally and why. If yes, ask if this is intentional, and how and why might they change priorities.*
	What advice would you give to another creative practitioner who is trying to expand the impact of their work to wider health inequities such as social, economic, and environmental issues?

### 2.2 Participants

Participants were recruited for the survey using purposive and snowball sampling where organizations were contacted to make referrals and survey respondents recommended other individuals. Inclusion criteria comprised adult practitioners working in the UK offering environmentally and socially engaged community programs that involved creative and cultural activities leading to health and environmental outcomes. Of 43 potential participants identified through 73 organizations, 19 practitioners (7 employees, 12 freelancers; 16 females, 3 males) completed the survey. Of these, 15 indicated a willingness to be interviewed and eight (4 employees, 4 freelancers; 7 females, 1 male) met the inclusion criteria. Reasons for exclusion involved interest but lack of practice in the research topic. Participants were paid £50 for taking part in the interview.

### 2.3 Materials

Materials comprised a privacy statement; participant information sheet; consent form; an online survey and online interview protocol.

### 2.4 Procedure

Low risk ethical approval was obtained for the study. Libraries, museums, galleries, heritage sites, gardens, and mailing list subscribers across the UK, contacted through CHWA, London Arts and Health, Happy Museum Project and Culture Declares Emergency, were sent recruitment emails containing project information and a survey link. The survey was conducted over 3 months (April–June 2023) using Qualtrics and took 10–15 min to complete. After the survey, participants were asked if they would like to take part in a one-to-one follow-up interview. For those who agreed, responses were screened for fit with inclusion criteria. Approximately 1 month after survey completion, interviews of c.60 min were conducted in Microsoft Teams over 2 months (May–June 2023).

## 3 Results

### 3.1 Question 1: Could you summarize your practice and the work that you do?

Responses to Question 1 were tabulated with practitioner quotations to illustrate their work (Table 3).

**Table 3:** Summary of practice and practitioners’ work.

Employed/ freelance	Summary of practice	Quotations illustrating practitioners’ work
Employed	Family learning programmer for museum and charitable trust	“Something that’s important is around welcoming and creating spaces where people can be themselves… and we meet them and then we ask questions to build on their confidence and curiosity and playfulness that goes across all the programs. And making that space for some empathy to develop. I’m really keen that… we are connecting to ourselves… and then we start to reach out and connect to others.”
	Wellbeing project coordinator for museum	“What we did was we set up a weekly event that was to connect people socially… we set up activities where people would just be busy doing something alongside somebody and easily have a chat as they did it. So, they did not just have to come in and sit and chat to people, but the activities gave a vehicle to be relaxed socially together, and it’s worked very well actually, we have had a really good response to it.”
	Primary care practitioner and researcher	“I calculated a few years ago, that I’d listened to more than 50,000 patient stories… what I think is that people do benefit from having a creative space to be heard and they often then have the solutions to the problems that they bring themselves. They just need to be heard and sometimes prompted and, if people have a space which feels non-judgemental, often they remember their own creative solutions to health.”
	Creative practitioner and university researcher	“I got interested in philosophy and arts from China and the East, Asia, and began learning and practizing and becoming immersed in what I now call Chinese embodied culture. The felt physical practice is a wisdom culture so it conveys a lot of meaning in terms of ecologies of being with the kinship of the land and the elements and with nature and of course with other people.”
Freelance	Arts and wellbeing practitioner for mental health charities	“It’s an arts-in-nature program about being creative outdoors, we believe that children need to spend time outdoors to build nature connectedness… It’s very much about it being child led and process led so in a nutshell a lot of the work we have being doing is about supporting children and young people to have these opportunities but then working alongside schools for them to build their capacity for this work.”
	Community arts practitioner and director of not-for-profit organization	“We facilitate community engagement in a coastal environment… what we do is always multi-disciplinary, that’s the aim. It’s arts and culture and heritage and science and wellbeing so we are not saying that one way of knowing, understanding, or exploring the coast is better than another, memories, perceptions, and sensory experience are just as important as citizen science.”
	Arts and heritage consultant and occupational therapist	“I’m with the clients and stakeholders co-developing programs that encompass a variety of things to do with art, nature, culture and heritage, and health and wellbeing. Then sometimes with museums working with specific populations, for example, people living with dementia and looking at the archives and resources and signposting, so it’s quite fluid in a sense.”
	Creative and mindfulness practitioner in nature connection	“A typical session might involve a friendly gentle welcome, cups of tea and asking everybody to talk about something they have noticed in nature in the last week or to bring them into the space and connect them back to where we left off the week before… we looked at movement in nature and used the movement of our bodies to connect with the pace of movement in nature.”

**Figure 1:**
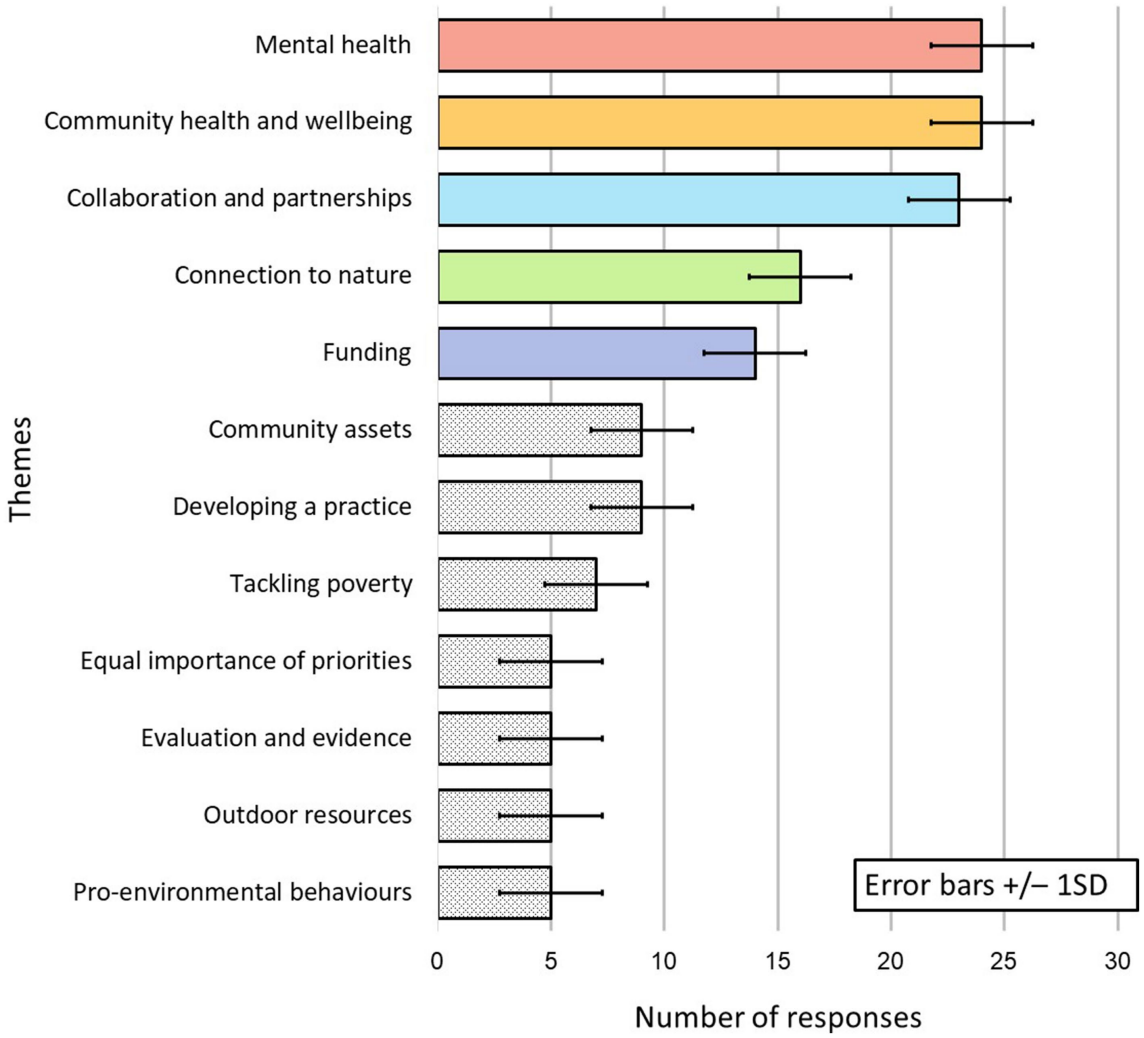
Number of responses to each theme.

For data from Questions 2–9, 12 themes were identified from 98 subthemes derived from 146 responses, mean number of responses per theme = 12.17 (median = 9; range = 19) and mean number of subthemes per theme = 8.17 (median = 7; range = 11). Five themes, with the number of responses above the mean with just under 70 per cent (69.18%) of all responses were regarded as major themes: ‘Community health and wellbeing’; ‘Mental health’; ‘Collaboration and partnership’; ‘Connection to nature’; and ‘Funding’. Seven themes with the number of responses below the mean were regarded as minor themes: ‘Community assets’; ‘Developing a practice’; ‘Tackling poverty’; ‘Equal importance of priorities’, ‘Evaluation and evidence’; ‘Outdoor resources’; and ‘Pro-environmental behaviors’ (Figure 1).

### 3.2 Question 2: What outcomes are you hoping for and how do you know if you have achieved them?

Five major and two minor themes were derived in response to this question (Table 4). Within ‘Community health and wellbeing’, these outcomes included supporting isolated and lonely people, creating a sense of safety and being welcome, connecting people, and practitioners looking after their own health as the first step. Practitioners determined whether outcomes were achieved by looking at attendance numbers and regularity, whether people felt safe, welcome and confident, and by using wellbeing frameworks such as the Community Spirit Framework. Within ‘Collaboration and partnerships’, intended outcomes involved trust, building networks, integrating with other initiatives, and sharing experiences. Practitioners determined whether outcomes were achieved by assessing whether participants were able to collaborate and co-produce across the sector and influence the wider systems. Within ‘Connection to nature’, the intended outcome was to connect with nature, respect nature and appreciate they were part of nature. They felt it was important for children and young people to access green spaces and for connection to nature to be built into school curricula, though the only outcome measure was to assess whether this connection had occurred long-term. Within ‘Mental health’, practitioners spoke about the impact of environmental concerns on mental health, but also the need to change the mental health system and move toward a model that was closer to their own creative approach, emphasizing prevention and the reduction of escalation. They discussed the need for better and quicker access to mental health services, and for services co-produced with children and young people who might themselves offer ideas about the support they required. Within the minor themes of ‘Community assets’ and ‘Evaluation and evidence’, participants stressed the importance of equitable access to community assets that might determine health; and said that evaluation should be “meaningful” and used as a “reflective tool.”

**Table 4:** Outcomes and knowing if they have been achieved: themes and subthemes.

Themes	Subthemes (no. of responses*)	Examples of practitioner quotations
Community health and wellbeing	Supporting isolated people (4)	“Outcomes around supporting people who are isolated and lonely and have mental health concerns and physical health concerns and boosting wellbeing … not only individual but community wellbeing as well.”
	Building confidence (2)	“A lot of the time what I strive for in my work is the sense that people feel more confident and comfortable in navigating spaces that are already on their doorstep.”
	Feeling safe and welcome (2)	“The outcomes that I’m looking for is that people notice for themselves but also feel safe, welcome and at home and that their curiosity, their play, their imagination are being enlightened, are fired up, are being given a space to be.”
	Wellbeing frameworks (2)	“We are looking at community wellbeing as well, so there are frameworks that we are following like, for example, the community spirit framework that the Royal Society of Public Health have published some information on.”
	Connecting people (1)	“One of our outcomes was to get people out and about and meeting together, so obviously, you know if you have achieved it by sheer volume of numbers coming in, and also the regularity with which people were coming.”
	Own health (1)	“The outcome that we were going for was to increase awareness that taking care of your own health is probably the first step, maybe the most important step, in terms of taking care of the planet. And that was our main outcome.”
Collaboration and partnerships	Influencing wider systems (3)	“It’s about a how we work as a group of small community organizations with charitable spaces, really grassroots, in order to influence that wider system.”
	Partner and stakeholder experiences (2)	“You’re having different partners and stakeholders bringing their experience; people with lived experience and people with professional experience can come together.”
	Practitioner networks (2)	“… looking at how can we work horizontally with other people and build networks of practitioners in a peer-to-peer kind of way you know.”
	Building trust in long-term relationships (1)	“There’s something about building that trust in long-term relationships with people, in the local community who come along to things and who also want to and like to have their say in the types of projects.”
	Co-production (1)	“Where people are connected in a sense, and you know people are aware and collaborating and co-producing together across the sector.”
	Integration with other initiatives (1)	“We have a lot of hope in terms of how this work integrates with other initiatives within local authorities so things like integrated care systems and the provision that the public health run to provide for schools.”
Connection to nature	Connection to nature in children (1)	“The overarching ambition is that children and young people get more time to build nature connectedness in formal education, and in practice that’s how we know it’s working from some of those long-term relationships.”
	Relationship with natural world (1)	“Like the kinship that I mentioned – that our health and wellbeing is so dependent on our relationships, not only with other people but with nature, with the earth.”
	Humans as part of nature (1)	“So, our innate self, you know, in connection with nature because we are nature, it’s within us and without us you know, and for me this is important.”
	Respect for nature (1)	“The impact of the climate crisis through flood and fires. You know nature is not our friend, it really needs respect and [we need] to work with nature.”
Mental health	Effects of the environment (2)	“You know in certain environmental challenges there are also issues with health and wellbeing that I think are related, everything’s related, but particularly around mental health issues that can impact on us.”
	Preventative measures (2)	“System change in terms of mental health and wellbeing, support from something that is about dealing with an escalation to moving to something which is much more preventative.”
	Support for children and young people (1)	“… how we make changes to the mental health system so that children and young people can get better access quicker as well as about how children and young people can co-produce what that mental health support looks like”
Community assets	Equitable access (2)	“Who feels comfortable around taking up space in places like parks, like beaches? Absolutely fraught with the kinds of barriers that are challenging people’s lives… it’s not an equitable thing, even if it’s a public space.”
	Cultural assets (1)	“And I think there’s something bigger there around the value of creativity and access to cultural assets being important for our health again.”
Evaluation and evidence	Evaluation as a reflective tool (1)	“We just do a sticker chart at the end and there’s circles and people can put them all in one circle or one in each… I think evaluation can be a really reflective tool to help the participants understand why we are here, what we are doing.”
	Meaningful evaluation (1)	“The whole thing with evaluation is that it has to be meaningful for the people filling it in as well as the people reading it.”
Total themes = 6	Total subthemes = 23 (36)	

*Similar responses made by different participants; similar responses made by the same participant only counted once.

### 3.3 Question 3: What key resources and spaces does your work make use of and why are they important to your work?

Responses to the question involved two minor themes (Table 5). Within ‘Community assets’, buildings needed to be accessible and offer warm space/hot meals during the winter. Practices involved “dovetailing with other initiatives” and working with anchor organizations, ideally having similar green policies, with a view to sharing resources. It was felt important to meet communities “where they are.” Within ‘Outdoor resources’, practitioners used green and blue, and rural and urban spaces. These resources were vital to some practitioners, but they faced challenges in applying for access to council-owned outdoor spaces and overcoming public transport issues in rural areas.

**Table 5:** Key resources and spaces and importance to work: themes and subthemes.

Themes	Subthemes (no. of responses*)	Examples of practitioner quotations
Community assets	Accessibility of buildings (1)	“It’s a fantastic building, it’s really accessible, we have got a lift up to the first floor for people who have got limited mobility, there’s no steps in the building as it’s been recently refurbished; it’s absolutely great as an accessible space.”
	Anchor organizations (1)	“So, you are in a town or a village and you are working with an anchor organization like a church or a community center or a library, so that’s a community space where you can work together going forward.”
	Community use of buildings (1)	“Throughout the winter months, the museum itself goes onto limited opening… what it means is the building is in use for the community during the winter. And that includes the café, and all the resources”
	Dovetailing with other initiatives (1)	“You can dovetail with other initiatives like the Men’s Shed Movement or just generally green initiatives, and I think that is something that people could recognize and replicate in their own environment, in their own community.”
	Meeting communities where they are (1)	“I think it’s important to go to the community as well… sometimes you need to meet the community where they are and we have done that, we have worked in the community, in their green space, or in their community hall.”
	Shared resources (1)	“Building relationships for shared resources. Shared resources and ideas about working together for the community because often they might have very similar green space policies nationally, for example like libraries.”
Outdoor resources	Access (1)	“There’s a space which is a community space for people to have access to, but you have to have a key for it, and you have to apply to get the key… so actually having access to a wild green space is like working in a very hyperlocal way.”
	Coastal environments (1)	“Of course, the beach is absolutely key. I live in Great Yarmouth so the beach that we tend to visit is Great Yarmouth beach, but we have also worked up in North Norfolk and we have also worked on the Suffolk Coast.”
	Green spaces near schools (1)	“One of the community assets that we were looking at was actually green spaces within schools or slightly beyond schools and supporting schools to better access those green spaces.”
	Urban areas (1)	“I’ve done nature connection sessions in a carpark or in very built-up urban areas, so the spaces that I need are basically outdoors, any time of year, anytime of day or night, outdoors.”
	Rural areas and transport issues (1)	“You can come on public transport but it’s a challenge, there’s not a huge amount and there’s no trains, there’s only buses and there’s not many buses and that’s beyond our control so we work with our early help team which is a council team, and we do projects.”
Total themes = 2	Total subthemes = 11 (11)	

*Similar responses made by different participants; similar responses made by the same participant only counted once.

### 3.4 Question 4: What is your project’s value/impact within the local community context?

Responses to the question involved two major themes (Table 6). The first was ‘Community health and wellbeing’ whereby practitioner offered resources to care agencies working in the community and, also helped people develop tools to support their health “for the rest of their life” while encouraging ‘spin-off projects’ that could continue. Within ‘Funding’, practitioners felt additional funding would allow them to do more to impact the local community—while noting that strategic, hyperlocal and partnership approaches maximized value for money.

**Table 6:** Project’s value/impact within the local community context: themes and subthemes.

Themes	Subthemes (no. of responses*)	Examples of practitioner quotations
Community health and wellbeing	Connecting with care agencies (3)	“We’ve linked up with the agencies that offer social care and dementia care in the community and what they are also recognising is, not just that we are a resource to help them connect with people with dementia or social needs in the community, we are also a building they can use.”
	Spin-off projects (2)	“Where a program then spins off some other project work where the group have forged really good relationships and then they go off and they actually want to continue by themselves in a creative capacity.”
	Own health (1)	“What we were trying to do was say to people in the village we are spending all of this carbon on your medication to help your health, but what would help the planet more would be if you improved your health… by walking.”
	Enabling the community (1)	“…trying to facilitate them in saying how they wanted to be fitter for people and planet rather than me telling them what would be better… I felt that I was trying to enable the community… to make their own decision about it.”
	Giving people tools for life (1)	“we are trying to give people the tools for the rest of their life, it’s not like you can only come here and only do this when you are here, that’s not true health. Looking after our health, it is important to do that every day.”
	Legacy (1)	“We are setting up as a legacy of the project a website which will be like a notice board for the village which will hopefully keep running by itself…”
Funding	Hyperlocal approaches (1)	“Taking a much more hyperlocal and partnership approach… is crucial for us when there are limited financial opportunities, and we are all small teams working often at capacity.”
	Impact within funding structure (1)	“I think we do as much as we can within the funding structure that I have locally, but there’s always more that we can do, and we do have dreams of ways that we could have more of an impact on our local community.”
	Strategic approach to limited resources (1)	“We’re trying to take a strategic place-based approach in terms of how we approach the limited resources available in terms of financial resources… what assets do we have as a community and how might we approach that in a more strategic way rather than competing.”
Total themes = 2	Total subthemes = 9 (12)	

*Similar responses made by different participants; similar responses made by the same participant only counted once.

### 3.5 Question 5: How do you frame your work in relation to climate change/planetary health?

Responses to the question incorporated two major and two minor themes (Table 7). In ‘Connection to nature’, practitioners encouraged participants to consider how changes in nature—weather and seasons, for example—might relate to their own lives/ Practitioners felt it was critical to help people, especially children and young people, notice the natural world to become more emotionally connected with it and therefore to care about it. Practitioners suggested that they were guided by research evidencing the beneficial effects of nature on wellbeing, and associated connection to nature with altruistic and positive environmental and social choices. They also noted, however, that individuals felt a “massive burden of responsibility” for global warming, despite this being more realistically the responsibility of governments and multi-national companies. Within ‘Funding’, one practitioner suggested funders could prioritize projects concerned with nature and embedded in local partnership. For minor themes, ‘Pro-environmental behavior’ included being “mindful” about resources such as lighting, packaging and recycling, and buying locally—with one practitioner explaining how their organization had taken part in carbon literacy training and set up an environmental working group. Within ‘Evidence and evaluation’, one practitioner suggesting there was “lots of robust evidence” on access to nature and pro-environmental behaviors, and creativity and health. Another referred to the Wheel of Wellbeing, a measure including care for the planet.

**Table 7:** Framing work in relation to climate change/planetary health: themes and subthemes.

Themes	Subthemes (no. of responses*)	Examples of practitioner quotations
Connection to nature	Changes in nature (2)	“We have adult mindful drawing events on all the solstices and equinoxes and that’s thinking about the turn in the seasons, how things change.”
	Connection to nature in children (2)	“A lot of our work is around how children and young people need to have a connection with nature to build the behavior that will support them to make decisions based on them having a connection to the natural world.”
	Burden of responsibility (1)	“Individual humans do feel a massive burden of responsibility for global warming; all these massive issues are actually the responsibility of governments and multi-national companies and so it’s something I go in circles a bit with.”
	Relationship with natural world (1)	“People who are more nature-connected make more altruistic decisions, make more positive decisions around environmental choices, around social choices, there’s this thread between being more nature-connected and feeling like you have got a relationship with the environment around you.”
	Emotional connections to natural world (1)	“Encouraging people to notice, and when they notice they become more emotionally connected, and when you are more emotionally connected to the natural world, it becomes more important to you, and that’s when you want to protect it.”
	Helping people to notice nature (1)	“I help people to see and feel and notice and begin to care about the natural world, and people talk about there being some process a bit like a magician pulling a rabbit out a hat. Something wasn’t there and then they have done an activity and suddenly they can see something they did not see before.”
Pro-environmental behaviors	Sourcing resources locally (2)	“We made a decision at the start of the project that we would source as many of our resources as locally as we could. So that’s a value that we stick to. We’ve done things like recycled crafts and things like that.”
	Carbon literacy training (1)	“We’ve all done carbon literacy training, and we have an environmental working group…”
	Mindful with resources (1)	“We’re always mindful on how we use resources, and being careful with the planet, and looking after the ground that we are walking on. And we give a space to hold some of those conversations.”
	Lighting, recycling, and packaging (1)	“We’ve changed all of our lighting, we looked at all of our recycling, all of our packaging so the real tangible things that we can do as well.”
Evaluation and evidence	Guided by research evidence (1)	“In recent years there has been a massive explosion in research around connecting with nature benefits for wellbeing… thousands of papers have come out about all kinds of aspects, and the same with creativity and health, there’s loads of robust evidence for all kinds of different practices.”
	Wheel of wellbeing (1)	“There’s a wheel of wellbeing… sometimes called WoW, a bit like the ‘five ways to wellbeing’ but care of the planet is another segment in it. In fact, the trust I used to work for, South London and Maudsley, they initiated this wheel of wellbeing.”
Funding	Financial support for nature projects (1)	“Initiatives that might help financially support projects that are looking at nature, so looking at drawing upon funds that help with that and combining it with partnerships, with the library or with a voluntary organization that are keen on greening their space or nature on prescription initiatives.”
Total = 4	Total = 13 (16)	

*Similar responses made by different participants; similar responses made by the same participant only counted once.

### 3.6 Question 6: What health/wellbeing issues does your work aim to address? What does tackling these issues look like in practice for you?

Responses to the question consisted of two major themes and one minor theme (Table 8). ‘Mental health’ incorporated the majority of responses to this question. Here, practitioners looked to research evidence, as with Question 5, citing that less time in green spaces was associated with a greater incidence of depression. Practitioners advocated interventions where participants were distracted from other concerns and encouraged to put their “worries to one side for a little while.” They felt that negative emotions would reduce through their interventions. Practitioners reported helping people who were lonely and isolated to reconnect, making spaces feel inclusive, giving their participants choices. They thought there should be preventative measures and a more equitable mental health system for children and young people, with ‘mental health literacy’ included in education; they pointed to evidence of the particular challenges experienced by people with protected characteristics. Practitioners also considered it important to look after themselves and their staff, pointing out the risk of stress associated with working mental health. Within ‘Community health and wellbeing’, practitioners spoke about the need to work with community groups, in one case saying that health inequalities can “affect everybody.” Within ‘Evaluation and evidence’, one practitioner explained that project timescales, and the challenge of knowing what to look for, made it “tricky” to assess wellbeing.

**Table 8:** Addressing and tackling health and wellbeing issues: themes and subthemes.

Themes	Subthemes (no. of responses*)	Examples of practitioner quotations
Mental health	Research evidence (3)	“We know a lot of the research that’s taken place recently, we have seen some really good reports around [young] people who spend less time in green spaces are more likely to have symptoms of depression in moving to adult life.”
	Distraction from worries (2)	“You can get some respite and some rest; you can put those things down for a little while and then you can pick them back up when you need to think about them again but let us see what it’s like to put those worries to one side for a little while.”
	Looking after our staff (3)	“But one of the things we are also mindful of is us and our staff because we need to really look after everybody as well because, otherwise, we cannot do our jobs well.”
	Mental health literacy (2)	I think there’s concern around how much people know about mental health factors, they probably tend to know quite a bit about what might be good for our physical health, but maybe not so much about mental health and wellbeing, so there’s a part of this that is to do with literacy in education.”
	Preventative measures (2)	“I think for some of these children it’s about if there aren’t preventative measures in place, they have to go through the system, it’s incredibly costly in terms of what council, local authority or NHS provide that they are going to have to go through.”
	Changing lives (1)	“Often in people’s lives I see things… if people did not have depression and anxiety, I think they would not really change their lives, so I think there’s an inherent creativity in disease.”
	Equity for children and young people (1)	“On the mental health side, it’s about wanting to build a more equitable system for children and young people… If your health is deteriorating and you are not able to attend school or you do not have wider support networks in place, there’s an inequity of access to both education and health.”
	Having a choice (1)	“Having a choice about what they want to do next, so I might just go ‘oh look at that amazing…’ I do not know, whatever ‘…bird, kestrel, color through the tree, oh wow look at that, how does that feel?’… and they can come back to where we are instead of all that stuff that’s going on in their heads.”
	Helping people step beyond their expectations (1)	“I think what I hear is that people say that they push beyond… they have been able to step beyond expectations of themselves, being able to creative and imaginative in ways that they did not really think they would be.”
	Improvement to negative emotions (1)	“I can definitely say that people who get referred through social prescribing to my sessions are often referred because of low self-esteem, poor mental health, anxiety, depression, poor sleep, those kinds of things, and they always talk about those things having improved through the course.”
	Making spaces feel inclusive (1)	“My aim is to make spaces that feel inclusive, welcoming and the way people can engage… there’s no expectation, there are different ways to be neuro-divergently inclusive; an engaged participant might be someone who’s head down and working on their own thing, it does not mean they are not gaining a lot from being there.”
	Protected characteristics (1)	“And thinking about protected characteristics as well, so we work with children and young people who identify as LGBTQ+ and a lot of the evidence there is really stark in terms of mental health challenges.”
Community health and wellbeing (2)	Health inequalities (1)	“We’re raising that as an issue, that health inequalities can affect everybody.”
	Working with community groups (1)	“We do lots of work with community groups, bringing community groups to the park, and wellbeing is at the core of that day.”
Evaluation and evidence (1)	Time scale (1)	“Evaluation is a tricky thing, what’s the timescale and what are you looking for? There’s a woman on the course… she had a baby in Week 3. Week 4 she arrived, this tiny baby strapped on to her, but she wants to stay on the course because what she’s learnt she’ll do with her child as it grows. How do you measure the benefit on that child’s wellbeing?”
Total themes = 3	Total subthemes = 15 (22)	

*Similar responses made by different participants; similar responses made by the same participant only counted once.

### 3.7 Question 7: What social and economic issues does your work aim to address and how does your work tackle them?

Responses to the question involved the major theme of ‘Funding’ and minor theme of ‘Tackling poverty’ (Table 9). Within ‘Funding’, practitioners stressed the importance of moving toward sustainable, long-term funding models. This would support respectful and timely payment for practitioners (and in some cases participants), project sustainability, and the development of a stronger evidence base. They also spoke about the “catalyst effect” whereby through increasing their knowledge and skills, community partners might develop the potential to apply for their own funding to sustain projects. Within ‘Tackling poverty’, practitioners noted that their activities were offered for free, and they were consciously working to support people to attend. Additionally, one practitioner suggested that the pro-environmental behaviors fostered by their work might also alleviate cost-of-living challenges, for example encouraging a culture of walking to school rather than driving.

**Table 9:** Addressing and tackling social and economic issues: themes and subthemes.

Themes	Subthemes (no. of responses*)	Examples of practitioner quotations
Funding	Paid practitioner opportunities (3)	“Striving to provide paid opportunities for people, so practitioners in the local area and also, people coming in as a participant, coming to a workshop as an assistant, coming as a facilitator… trying to do my best to find opportunities for people that are well paid – nice and quickly, and respectful of people’s time.”
	Sufficient funding (3)	“… in terms of our supply and short-term funding, being able to do a little bit of work and then not having the evidence base necessary for commissions from organizations like public health.”
	Longer-term funding (2)	“I think there needs to be acknowledgement from funders around longer-term funding and the full ecosystem.”
	Skills to apply for funding (1)	“There’s a bit of a catalyst effect hopefully so that people are not only being able to connect with each other but to reinforce the potential to sustain those projects so that they have more knowledge, more skills to apply for funding.”
	Sustainability of projects (1)	“I think the thing is around sustainability is that you might have a project that is funded but then how do you continue that funding?”
Tackling poverty	Offering activities free of charge (2)	“We offered all our activities free of charge so that really anybody could come… We are trying to keep that balance of being able to offer it to free to anyone who wants it and needs it, but also making it available in a targeted way and those who are able to pay can pay if they want to.”
	Addressing the cost-of-living crisis (2)	“In the cost-of-living crisis if there is a culture of walking to school instead of driving, it is something that could be universally accessed and cheaper.”
	Supporting people in poverty (2)	“Poverty is one of our key cornerstones of the work that we do so we are always trying to make sure that we are reaching out and being as mindful as possible and helping support people…”
	Working people facing challenges (1)	“We maybe have got two working adults in the household and have lots of things you need in life so they may have shelter, they may have food in the cupboards, they may have a choice of clothes to wear, they may have a holiday once a year, but they may also be facing some real challenges.”
Total themes = 2	Total subthemes = 9 (17)	

*Similar responses made by different participants; similar responses made by the same participant only counted once.

### 3.8 Question 8: Considering these different health, social and environmental aspects of your work, do you feel your work prioritizes any outcomes in particular?

Responses to the question produced one major and two minor themes (Table 10). Within ‘Equal importance or priorities’, most practitioners felt that the health, social and environmental aspects of their work were of equal importance. They saw these priorities as “intersecting,” “interweaving” and “multifaceted.” ‘Connection to nature’ included developing a relationship with and understanding and valuing the natural world and using art-making to foster empathy for different perspectives. Within ‘Community health and wellbeing’, one practitioner prioritized “the social side” with a view to building a sense of group and community “to get people together.”

**Table 10:** Priorities among health, social and environmental outcomes: themes and subthemes.

Themes	Subthemes (no. of responses*)	Examples of practitioner quotations
Equal importance of priorities	Intersecting priorities (2)	“I think that they all intersect in our work, and they bounce off one another, I would not say that there’s a priority in any one of those things, I think that they are all important.”
	Equal concern for human and planetary health (1)	“The two big projects I’ve been working on have encompassed natural heritage and therefore they encompass planetary concerns and the natural environment… I do think it has been equalized in a sense but equally how the health of our planet and people’s health fit within the environment, it does feel like there’s greater acknowledgment in that.”
	Interweaving practices (1)	“We had a new charitable objective about the environment, social change, and inclusion, looking at social justice and tackling inequalities. So, it’s not only that we are interweaving different artistic and somatic practices – no, we are interweaving different disciplines and cultures.”
	Multifaceted priorities (1)	“I think there’s a sense of it being multifaceted. Sometimes when I’m writing a funding bid for a project, they might emphasize this is particularly about social isolation or an environmental project… it’s absolutely a true picture of what we will do and what has happened before… but what I will say is that actually on the ground things are always multistranded.”
Connection to nature	Relationship with natural world (1)	“There’s always something around nature connection and developing a relationship with the natural world and something around learning species perhaps or learning different techniques in a more formal sense as well as in a more internal sensory sense.”
	Valuing the natural world (1)	“We have green space and blue space, we have animals, we have old trees, we have new trees, we have wild places, we have tidy places, we have wind, we have water, we have puddles. That is such a gift, and we know that it makes people feel better by being in that and it’s our duty to help people understand that and to value that…”
	Making space for safe conversations (1)	“Activities that we do to explore planetary health and the climate emergency. Often, it’s through creative practices, making space to have those safe conversations that could include emotions like fear, and confusion, and feeling sad, or feeling angry about decisions.”
	Different perspectives through art (1)	“Things that are really important to me at the moment are around empathy, I think we have a real privilege… we can build understanding of the world that we live in and help look at things from different perspectives through art.”
Community health and wellbeing	Connecting people (1)	“I think probably we have prioritized the social side of it most of all. Trying to help people to reconnect and building that sense of a group and a community you know to get people together to get people connecting well. I think probably, out of all of them that would be the one we have emphasized the most.
Total themes = 3	Total subthemes = 9 (10)	

*Similar responses made by different participants; similar responses made by the same participant only counted once.

### 3.9 Question 9: What advice would you give to another creative practitioner who is trying to expand the impact of their work to wider health inequities such as social, economic, and environmental issues?

Responses to the question involved one major and two minor themes (Table 11). Within ‘Collaboration and partnerships’, collaborating with other practitioners was seen as essential, particularly “interesting partners” and “like-minded small organizations.” Practitioners stressed the value of forming partnerships with funders, co-producing across the cultural sector, and consulting the community. They advised others to liaise with public health, build relationships with the NHS and local authorities, and connect with other agencies such as social and dementia care. Within ‘Developing a practice’ they spoke about the need “to grow some roots” within a particular discipline, without becoming “shackled by traditional practice.” They also talked about taking a variety of approaches to promoting the work and favoring inclusive language (“careful choice of words”) to ensure a breadth of participation.

**Table 11:** Advice to another creative practitioner: themes and subthemes.

Themes	Subthemes (no. of responses*)	Examples of practitioner quotations
Collaboration and partnerships	Collaboration with other practitioners (2)	“In partnership both organizations look at the in-between, we look at how to bring diverse practices together and we develop methodologies for collaboration between them when we interweave the roots of practice. What’s really interesting is the gaps and what can grow from the in-between us.”
	Connecting with other agencies (2)	“What I would recommend to people is connecting with other agencies. We connected with the college that was working with the adults with learning disabilities. We connected with that through dementia care… and with other agencies doing social care in the community… It stopped us duplicating anything and, also, it’s been mutually supportive.”
	Liaising with public health (2)	“Working across the board, liaising with public health… how public health is organized within local authorities, local councils. There seems to be a connection with museums and community engagement projects and libraries. So, there’s a hierarchy where public health is situated, from my experience.”
	Consultation with the community (1)	“I’d advise someone to really listen to what’s happening in the community that they are working in, so to be responsive… I think there’s something important about working in partnership, having a close ear to the ground, what it is that people want… and I think, in that respect, projects then are naturally very relevant and engaging because the practitioner consulted with the people.”
	Co-production (1)	“We need to look at the cultural sector, the museums, the libraries, the other arts organizations, to look at their ambitions and work together because it’s again about co-production.”
	Partnership with environmental organizations (1)	“I tap into what the National Lottery Heritage Fund is doing and Historic England because they are across the natural environment, our natural heritage and the wellbeing side of things… so you know it’s looking at how you can build the evidence to work in partnership with that ecosystem again basically.”
	Relationships with NHS and local authorities (1)	“I think building relationships with the NHS and the local authority and specific elements of the local authority is really crucial in order for you to understand what they need in order to be able to help you scale up your work.”
	Working with interesting partners (1)	I worked in partnership with some amazing environmental organizations. That was exciting, it felt dynamic, we were really learning… [it’s] easier in a way because I’m part of a team that has all that knowledge and expertise about the environment. That’s another top tip, find yourself some interesting partners.”
	Working with like-minded organizations (1)	“I think it’s finding out who are you, where you have a common language with other organizations. I think our journey has been about finding like-minded small organizations who are in some ways different, but our aims and overarching values are the same.”
Developing a practice	Not being constrained by practice (3)	“Although developing a root in a particular practice, not to become constrained by that. Sometimes with the practices that we do, particularly some traditional practices, we can become shackled by them so to really get into a practice that is liberational and not oppressive, keep an open mind.”
	Engaging with creative health (2)	“I think to engage with creative health… there’s lots of really interesting practices that are happening and it’s fantastic that there are programs that are bringing a more interdisciplinary way of how we consider health in a more social, wider sense and what we need as citizens to live well.”
	Choice of words (1)	“The other think I would advise is, the whole thing in your marketing, advertising, promoting of what you are doing, that careful choice of words. So, we have been careful to say this is not a dementia group, it’s not a mental health group but have used words that say: ‘dementia friendly’, ‘accessible’, ‘for all abilities’, and it’s meant that we have had a real breadth of people coming in.”
	Embodied practice (1)	“I would recommend a practice that’s embodied… and I would describe an embodied practice as one that involves the felt sense, so when we speak about mind–body, to think of other words for describing it like ‘psycho-physical’, so not something that’s purely cognitive or purely physical that I do not believe can exist.”
	Gaining experience (1)	“I think looking back on my career, I think it’s important to grow some roots in a particular practice to get some experience and really look into oneself through a particular practice.”
	Using different approaches (1)	“Some people do not necessarily access the internet and might not find it that way so therefore you need a variety of ways to promote what’s on offer, using different approaches, I think to reach people when there’s information in different ways, they might only notice something because they are sitting at a table and then they can see one of these adverts.”
Total themes = 2	Total subthemes = 15 (21)	

*Similar responses made by different participants; similar responses made by the same participant only counted once.

### 3.10 Common features

**Figure 2:**
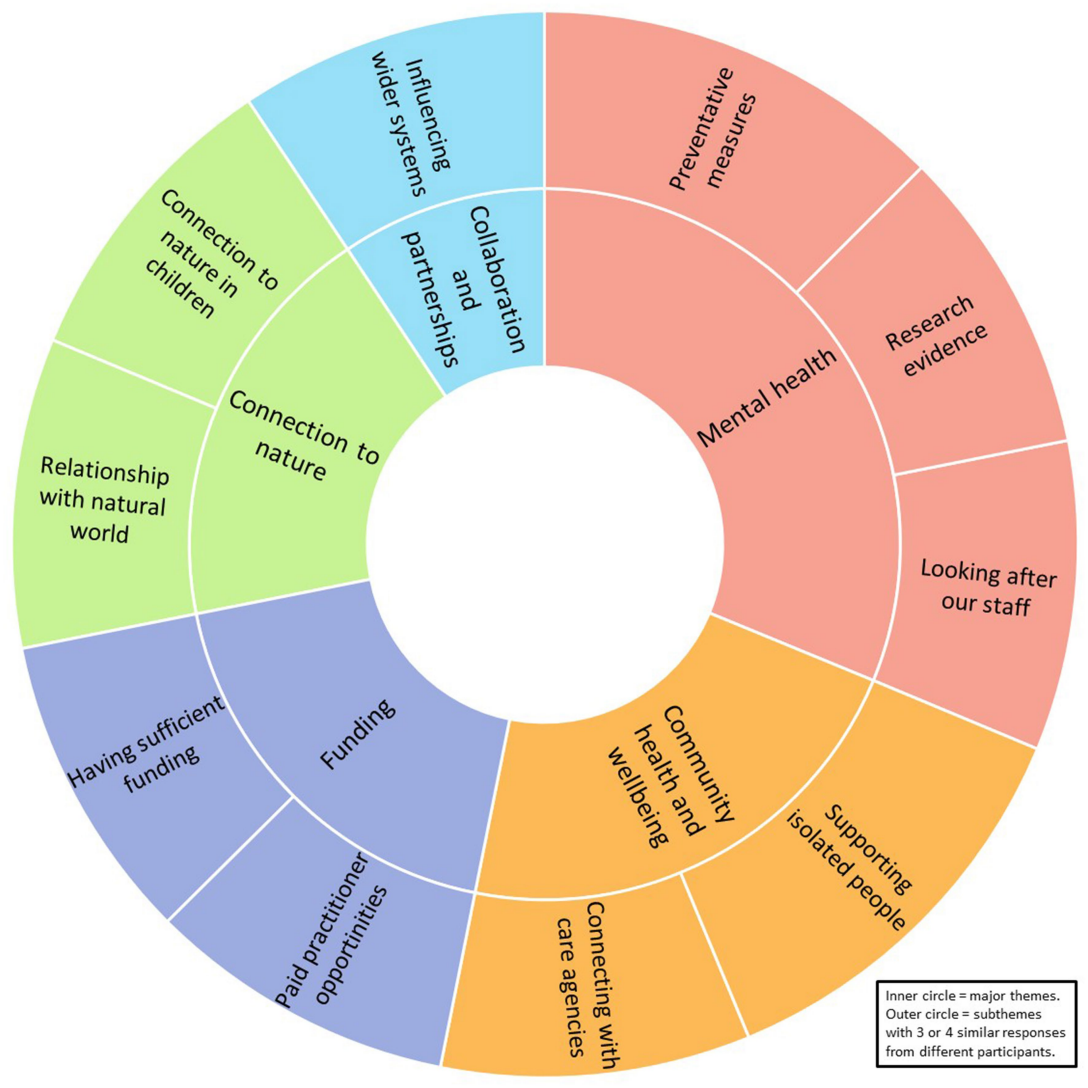
Common features of environmentally and socially engaged community programs.

To address the aim of identifying common features of environmentally and socially engaged community programs, subthemes derived from three or four similar responses (37.5–50% of practitioners) were located by combining Tables 3–11 (Appendix 1). Ten subthemes, derived from 32 responses constituting just over a fifth (21.92%) of all responses and contributing to the five major themes, were identified as common features of the practices of those interviewed (Figure 2). The two most common features, each with four similar responses, were ‘Preventative measures’, within the theme of ‘Mental health’ and ‘Supporting isolated people’, within the theme of ‘Community health and wellbeing’. The remaining eight common features, each with three similar responses, were ‘Research evidence’ and ‘Looking after our staff, within the theme of ‘Mental health’; ‘Connecting with care agencies’, within the theme of ‘Community health and wellbeing’; ‘Having sufficient funding’ and ‘Paid practitioner opportunities’, within the theme of ‘Funding’; ‘Connection to nature in children’ and ‘Relationship with the natural world’, within the theme of ‘Connection with nature; and ‘Influencing wider systems’, within the theme of ‘Collaboration and partnerships’.

## 4 Discussion

The current research aimed to identify common features of environmentally and socially engaged community programs, specifically those addressing the intersecting challenges of planetary and human health. To achieve this aim, the study analyzed survey and interview data from creative health practitioners. Practices employed a variety of interventions involving arts and crafts, breathing techniques, creative writing, discussion, forest bathing, gardening, meditation and mindfulness, singing, and walking. Some activities were held indoors whereas others took place in green and blue, and rural and urban spaces. Findings showed that over two thirds of participant responses were distributed across five major themes: ‘Mental health’; ‘Community health and wellbeing’; ‘Funding’; ‘Connection to Nature’; and ‘Collaboration and partnerships’. Ten of the subthemes within these major themes, based on three or more similar responses from different practitioners, were deemed common features of community programs (Figure 2).

On the whole practitioners felt that the health, social and environmental aspects of their work were of equal importance in that they were interconnected and needed to be addressed together. A study determining priority areas for future research into inequalities showed a similar finding (24). Practitioners acknowledged the intersection of planetary and human approaches to health, promoting the idea that arts engagement could help to “mitigate the effects of an adverse environment” [(30), p. 10]. Some practitioners, though, held individual views not shared with other practitioners. Consequently, of the 10 subthemes reflecting common features among practices, only two addressed the intersecting challenges of planetary and human health directly. These were ‘Connection to nature in children’ and ‘Relationship with natural world’, through practices recognizing environmental and human interdependency. Findings aligned with the work of other authors who hypothesized that connection to nature might mediate health and wellbeing gains from the natural environment that might play a role in pro-environmental or pro-conservation and behaviors (34). Evidence for these benefits, however, was described as “patchy” and recommendations aligned with the notion that findings should be “brought systematically together to inform transferal and scaling of programs between contexts and populations” [(32), p. 81].

Four subthemes of ‘Influencing wider systems’, ‘Looking after our staff’, ‘Preventative measures’, and ‘Research evidence’ addressed the intersecting challenges of planetary and human health indirectly through practitioner partnership influence over policies relating to climate change, and by addressing concern for the environment manifesting in eco-anxiety. Analysis of community practitioner case studies showed that connections needed to be built between climate and health through creative programming intended to link nature, health and wellbeing outcomes (37). Other researchers proposed that planetary health should draw attention to the “integration of biological, psychological, social and cultural aspects of health in the modern environment” [(10), p. 1], aligning with the biopsychosocial model of health (38). In the current study, practitioners talked about developing their practices over several years and typically described their work as “process-led with a focus on the doing of the activity” in contrast with evaluation of end products such as artworks or wellbeing. As a result, practices were not set up as models intended to build creative, climate and health connections Instead practices evolved over time and, as one practitioner expressed, “adjusted to prevailing trends.”

With respect to mental health, practitioners described their own feelings of anxiety toward climate crisis or those of their collaborators or participants. As one creative health practitioner stated, “I have a lot of tension in myself where I think that I should be doing more” and “individual artists do feel, and individual humans do feel a massive burden for responsibility for global warming… all these massive issues.” Along with the emotional impacts of working with people facing mental health challenges, this ‘eco-anxiety’ also contributed to the subtheme of ‘Looking after our staff’. Another practitioner explained how creative activities facilitated space for discussion on the “collective trauma” of climate change. Practitioners were keen to promote “system change in terms of mental health and wellbeing,” moving from “dealing with an escalation” to “something which is much more preventative.” Although they did not formally evaluate their own projects, practitioners commonly cited various sources, particularly mental health studies, advocating increased use of preventative measures such as creative health practices. They suggested that the natural environment could be used as a resource for the prevention or treatment of poor mental health and that longer-term programs might be more effective than those in the short-term. Participants highlighted the need for more good quality evaluation and research on the impacts and cost-effectiveness of natural environment-based health interventions. Their suggestions were in keeping with the proposal that “existing evidence should be brought together using systematic approaches” [(32), p. 81].

The remaining four subthemes informing common features of practice (‘Connecting with care agencies’; ‘Supporting isolated people’; Having sufficient funding’; and ‘Paid practitioner opportunities’) were ostensibly concerned with human health and wellbeing particularly in isolated communities. Common features of practice were underpinned by connecting with other people and agencies and obtaining funding. Practitioners were generally of the view that developing their own approaches and resources “strengthened local social, economic, environmental, cultural and political circumstances” [(20), p. 11]. Developing these approaches led them to boost both their own and community health (20). Nearly all practitioners reported that their intended outcomes related to addressing social factors aligning with statistics showing that 30–55 per cent of health outcomes are rooted in social outcomes which are “more important than health care or lifestyle choices in influencing health” [(28) p. 1]. Practices were in keeping with the assertion that “the most effective actions to reduce health inequalities will come through action within the social determinants of health” [(11), p. 86]. They agreed that in public policy discourse, engagement with the arts and culture is “typically connected to such wider societal targets as improving the welfare of citizens, building social connectedness, revitalizing marginalizing areas, and boosting creativity and innovativeness” [(27), p. 16]. The common features of obtaining sufficient funding and paying practitioners were unsurprising given the wider state of underfunding for both direct delivery and the ecosystem of the creative health sector (39). It is also worth noting that higher education institutions typically can apply for funding for research but not for service provision. Case studies exploring barriers and enablers faced by UK creative and cultural practitioners addressing planetary and human health found that a key barrier was a lack of long-term funding to develop capacity for partnerships, networks, and policy influence whereas a key enabler was institutional commitment to increase resources (37). Findings were, therefore, consistent with the case study evaluation in that “climate, health and creativity are not separate in people’s experiences” but they are separated by “funding and organizational structures” [(37), p. 1].

### 4.1 Limitations

Findings from the current study should be interpreted with caution because of the restricted number of practitioners interviewed due to the stringency of inclusion criteria and the fact that several practitioners who completed the survey did not consider their work to be creative or cultural despite being referred by another participant or organization. It should be noted that seven out of eight practitioners interviewed (87.5%) were female, typical of the creative health sector; diversity statistics for creative health found a similar female workforce (87.8%) (40). Demographic data was not sought, however, so age or ethnicity cannot be compared. Practitioners regularly reported that limitations on funding makes formal evaluation challenging, so evidence was not sought in the form of reports or evaluations; instead, anecdotal evidence reported by practitioners was analyzed. Consequently, views could be said to be subjective rather than objective, though in keeping with the constructivist methods used. A research approach only employing larger organizations with the funding, expertise, resources, and time to carry out robust evaluation would have risked excluding many creative health projects. In employing an interpretivist epistemology, the researchers understand that they are never removed from the research process and acknowledge that their understanding of the potential of creative health practices may have predisposed them to certain conclusions.

## 5 Conclusion

The current study aimed to expand upon the Creative Health Quality Framework (36) by identifying common features of environmentally and socially engaged community programs, specifically those addressing the intersecting challenges of planetary and human health. Creative health practices described by those interviewed were diverse, varying greatly depending on the communities in which they were located, and the cultural or creative organisations, practitioners and resources involved. The interventions, while superficially simple—often founded in conversation—rest on paying careful attention to bringing people together in spaces that feel open, safe and warm and being led by their own responses to nature and creativity. By identifying commonalities, the study drew attention to practitioners’ socially determined views of health, and desire to work collaboratively for mutual support and shared resources. Results of the study may be utilized by commissioners and funders to better understand connections between planetary and human health as they operate for community organizations and to consider how their funding and commissioning structures might support these. Results may also be helpful for creative practitioners to address the wider social determinants of health, including planetary health, to tackle health inequalities with the intention of maximizing “human wellbeing for present and future generations while remaining safely within ecological boundaries” [(2), p. 1]. To achieve this goal requires “a more complete understanding of community wellbeing and alternative ways of characterizing it” [(2), p. 1]. Given that until recently wellbeing was understood “mainly in economic terms,” it is now necessary “to enrich the understanding of wellbeing on the basis of a relational paradigm, in which the dependency of human wellbeing on the health of the ecosystems is internalized” [(13), p. 167]. As it is likely that creativity and culture are under-tapped resources, the potential to address community and environmental issues to tackle health inequalities, especially those resulting from climate injustice, has not yet been fully realized. The current study indicates the need for inclusive practice, partnership work, and sustainable funding which can support both practitioner wellbeing and the process, outputs and impacts of natural and sustainable environment-based health interventions and other resources instrumental in preventative healthcare.

## Data Availability

The original contributions presented in the study are included in the article/Supplementary material, further inquiries can be directed to the corresponding authors.
