# Supplementary material for: Common features of environmentally and socially engaged community programs addressing the intersecting challenges of planetary and human health: mixed methods analysis of survey and interview evidence from creative health practitioners
Source: Front Public Health. 2025 Jan 27;13:1449317. doi: 10.3389/fpubh.2025.1449317 (PMC11808534; doi:10.3389/fpubh.2025.1449317)
Supplement: Supplementary file 1 [file Table_1.docx]

Appendix 1. Questions 2–9: Themes, subthemes and number of responses

| Major/minor themes | Themes | Subthemes (number of responses*) |
| --- | --- | --- |
| Major themes | Mental health | Preventative measures (4) |
|  |  | Looking after our staff (3) |
|  |  | Research evidence (3) |
|  |  | Distraction from worries (2) |
|  |  | Effects of the environment (2) |
|  |  | Mental health literacy (2) |
|  |  | Changing lives (1) |
|  |  | Equity for children and young people (1) |
|  |  | Having a choice (1) |
|  |  | Helping people beyond expectations (1) |
|  |  | Improvement to negative emotions (1) |
|  |  | Loneliness and isolation (1) |
|  |  | Making spaces feel inclusive (1) |
|  |  | Support for children and young people (1) |
|  | Community health and wellbeing | Supporting isolated people (4) |
|  |  | Connecting with care agencies (3) |
|  |  | Building confidence (2) |
|  |  | Connecting people (2) |
|  |  | Feeling safe and welcome (2) |
|  |  | Own health (2) |
|  |  | Spin-off projects (2) |
|  |  | Wellbeing frameworks (2) |
|  |  | Enabling the community (1) |
|  |  | Giving people tools for life (1) |
|  |  | Health inequalities (1) |
|  |  | Legacy (1) |
|  |  | Working with community groups (1) |
|  | Collaboration  and partnerships | Influencing wider systems (3) |
|  |  | Collaboration with other practitioners (2) |
|  |  | Connecting with other agencies (2) |
|  |  | Co-production (2) |
|  |  | Liaising with public health (2) |
|  |  | Partner and stakeholder experiences (2) |
|  |  | Practitioner networks (2) |
|  |  | Building trust in long-term relationships (1) |
|  |  | Consultation with the community (1) |
|  |  | Co-production with cultural sector (1) |
|  |  | Integration with other initiatives (1) |
|  |  | Partnership with environmental organisations (1) |
|  |  | Relationships with NHS and local authorities (1) |
|  |  | Working with interesting partners (1) |
|  |  | Working with like-minded organisations (1) |
|  | Connection to nature | Connection to nature in children (3) |
|  |  | Relationship with natural world (3) |
|  |  | Changes in nature (2) |
|  |  | Burden of responsibility (1) |
|  |  | Different perspectives through art (1) |
|  |  | Emotional connections to natural world (1) |
|  |  | Helping people to notice nature (1) |
|  |  | Humans as part of nature (1) |
|  |  | Making space for safe conversations (1) |
|  |  | Respect for nature (1) |
|  |  | Valuing the natural world (1) |
|  | Funding | Paid practitioner opportunities (3) |
|  |  | Sufficient funding (3) |
|  |  | Longer-term funding (2) |
|  |  | Financial support for nature projects (1) |
|  |  | Hyperlocal approaches (1) |
|  |  | Impact within funding structure (1) |
|  |  | Skills to apply for funding (1) |
|  |  | Strategic approach to limited resources (1) |
|  |  | Sustainability of projects (1) |
| Minor themes | Community assets | Equitable access (2) |
|  |  | Accessibility of buildings (1) |
|  |  | Anchor organisations (1) |
|  |  | Community use of buildings (1) |
|  |  | Cultural assets (1) |
|  |  | Dovetailing with other initiatives (1) |
|  |  | Meeting communities where they are (1) |
|  |  | Shared resources (1) |
|  | Developing a practice | Not being constrained by practice (3) |
|  |  | Engaging with creative health (2) |
|  |  | Choice of words (1) |
|  |  | Embodied practice (1) |
|  |  | Gaining experience (1) |
|  |  | Using different approaches (1) |
|  | Tackling poverty | Addressing cost-of-living crisis (2) |
|  |  | Offering activities free of charge (2) |
|  |  | Supporting people in poverty (2) |
|  |  | Working people facing challenges (1) |
|  | Evaluation and evidence | Evaluation as a reflective tool (1) |
|  |  | Guided by research evidence (1) |
|  |  | Meaningful evaluation (1) |
|  |  | Wheel of wellbeing (1) |
|  |  | Time scale (1) |
|  | Outdoor resources | Access (1) |
|  |  | Coastal environments (1) |
|  |  | Green spaces near schools (1) |
|  |  | Urban areas (1) |
|  |  | Rural areas and transport issues (1) |
|  | Equal importance of priorities | Intersecting priorities (2) |
|  |  | Equal concern for human and planetary health (1) |
|  |  | Interweaving practices (1) |
|  |  | Multifaceted priorities (1) |
|  | Pro-environmental behaviours | Sourcing resources locally (2) |
|  |  | Carbon literacy training (1) |
|  |  | Mindful with resources (1) |
|  |  | Lighting, recycling, and packaging (1) |
|  | Total themes = 12 | Total subthemes = 98 Total responses* = 148 |

*Similar responses made by different participants; similar responses made by the same

participant only counted once.
